# The Sanitary Regulations of Antient Rome
*Galeni Opera. Edit. Kühn.Aretæus, Opera omnia. Edit. Kühn.Celsi (A. C.) De Medicinâ, libri octo.Horatii (Quinti Flacci) Opera.Cicero (M. T.) De Republicâ.Lyell (Sir Charles). Principles of Geology.Clark (Sir James, M.D.). On Climate.


**Published:** 1855-06

**Authors:** Alfred Haviland


### THE SANITARY REGULATIONS OF ANTIENT ROME.*

The numerous sanitary regulations in force at Rome, previous to its decline and fall, were instituted mainly to meet certain peculiarities in the site of this ancient city. Some account is therefore necessary of the physical and geological characters of this site before the expensive regulations of the various Roman governments to provide for the health, comfort, and welfare of their people, can be duly appreciated. We shall, therefore, consider—

I. The site of antient Rome, its physical and geological peculiarities, its climate, its common diseases, and its most remarkable epidemics.

II. The regulations for personal cleanliness, supply of water, aqueducts, and baths.

III. The regulations for the cleanliness and ventilation of houses and streets, drainage, windows, sleeping apartments, doors, height and aspect of dwellings, number of houses and inhabitants.

IV. The means used to keep the physical powers of the Romans as perfect as possible. Exercises, games, marriage, employment of time, construction of large buildings, and exercises in the army.

V. The means used to ward off epidemics. Religion, physicians, Esculapius, &c.

VI. Mode of burial, &c.

*Site of antient Rome.* In studying the means resorted to by the inhabitants of a city for the purpose of preserving their health, or providing for the daily necessaries of life, due regard must be paid to the several conditions under which the city may be placed. All cities or towns do not enjoy the same advantages either as to site, aspect, soil, water, or climate. Every city and town enjoys some individuality. This individuality may consist in the material of which the houses are built, and it is interesting also to observe the transitional character of the buildings of an old town, in whose neighbourhood the resources for construction have been gradually developed.† Originally the palace of the rulers and the hut of the artisan and slave were built of the same materials, and the same state of things may obtain in the future construction of yet unborn cities. From this consideration of the dwellings of a city, the mind

---

* Galeni Opera. Edit. Kühn.
Aretæus, Opera omnia. Edit. Kühn.
Celsi (A. C.) De Medicinâ, libri octo.
Horatii (Quinti Flacci) Opera.
Cicero (M. T.) De Republicâ.
Lyell (Sir Charles). Principles of Geology.
Clark (Sir James, M.D.). On Climate.

† The material with which the Cloaca Maxima was built indicates the antiquities of this town, for it is not the *peperino* of Gabri and the Alban hills, but the *tufa bitoide* of Brocchi. Smith.

is naturally led to the geology of its site; this leads us to reflect on the character and amount of the supply of water, and so on imperceptibly from one peculiarity to another, until at last a good general idea is obtained of the circumstances under which the people living in any given locality are placed.

We shall, therefore, treat on each element of the sanitary condition of antient Rome separately, and then review them in a collected form, and compare the effects of each individual influence on the health of the people who were once masters of the world, and whose city gave a name to an empire which represented the bodily and mental vigour of those who reared and sustained it.

*Physical Characters.* The seven hills, on which antient Rome was built, were the boundaries of an alluvial plain through which the Tiber has bent its course without much alteration from the earliest period of its history. This river describes a figure, in its route from the north to the south, resembling the letter S; the northern bend, directed towards the west, had consequently its concavity looking to the east, and embraced a plain, a portion of which was the celebrated Campus Martius, which was bounded by the Pincinian, Quirinal, and Capitoline hills: these boundaries gave the space an irregularly quadrilateral form. This plain, however, is only a portion of a more extensive one, from which arise the other four hills to the east and south-east of those just mentioned; thus there was to the east the Esquiline, and south-east of this hill the Cœlian, to the south-west of which was the Aventine, all which with the Capitoline formed an irregular circle around the Palatine, where according to the legend Rome was once but a small castle and gave a refuge to the outcasts of the neighbouring countries, who at last became so numerous that they covered the encircling hills with their habitations before the death of the founder took place. That part of Rome, bounded on the east by the southern bend of the Tiber, also is situated on a plain, whence arise the hills Janiculum and Vaticanus. Thus we see Rome was divided by the Tiber into two unequal parts; the western district was, however, only a suburb, and enclosed by the walls of the city, which were built of brick. The flat which lay at the feet of the hills of Rome was frequently inundated by the Tiber, on whose banks no quays were built nor works raised in order to keep out the intruding waters. And as the level of the river above that of the sea, into which it empties itself, was only thirty-five or forty feet, any circumstance retarding this current, as a long-continued westerly wind, caused the water to accumulate until a flood ensued. It is remarkable that the Romans never provided against an accident which must have materially increased the unhealthiness of the neighbourhood. These floods are alluded to in the story of Silvia, the Vestal virgin, who was seduced by Mars. The god, taking advantage of a total eclipse of the sun, to ruin the maiden in a cave, where she had fled to save herself from a wolf. Twins were the result of this amour, which, whether by design or accident, were washed by the flood to the foot of Mount Palatine, and there overturned by the root of a fig-tree near the den of a she-wolf, who carried them in and suckled them. These inundations, and the mention of a fig-tree, remind us of what Horace said about the heat of summer bringing "fresh figs and funerals," owing to the malaria which then arise.

The hills of Rome, whereon the flocks of the neighbouring Latin, Etruscan, and Sabine shepherd were once fed, whereon these people held councils of war one against the other, were separated by swampy valleys, in which many a desperate struggle took place in the time of war, and the friendly *comitium* during peace. Insignificant in height, however, as these hills were, they were sufficient to raise the people who dwelt upon them to a comparatively healthy height above the swampy plains of the yellow Tiber. The Esquiline, supposed to be so named from its thickets of oak (*æsculeta*), is the loftiest, being 218 feet high. Continuous with it are the Viminal and the Quirinal hills, the former of which derives its name from the thickets of osiers (*viminum sylvæ*) which grew there, and are significant of the marshiness of the neighbourhood. Mount Palatine, which has a central and somewhat isolated position, is 170 feet high. The Capitoline, which is the lowest, being only 150 or 160 feet, is however remarkable on account of its sides being more precipitous than those of the others, and from not being lessened in declivity by rubbish and broken rock.

*Geological Character.* Rome rises from a district which seems to have been the site of repeated volcanic disturbances. The springs of the Campagna are still found to be unwholesome, in consequence of their holding in solution excess of carbonate of lime and sulphuretted hydrogen. The beds of ancient lakes have been discovered in the neighbourhood of the city. Thus, the Ponte Leucano, in all probability, was suddenly dried up by an earthquake; the Travertin basin, however, which had been accumulating for ages, being left to tell the tale and afford material for the construction both of antient and of modern Rome; for the most splendid edifices of this city were built of the stone quarried on this spot and brought down by the river Anio, which is navigable from Tibur to its confluence with the Tiber.

In modern times the same processes seem to be going on in the now-existing lakes, for between Rome and Tivoli is the lake of Solfatara, (Lacus albula, Lago di Zolfo, Aquæ Albulæ of Galen,) which receives a tepid stream of water from one above it; and Sir H. Davy, who is quoted by Lyell, said of it, "I have found by experiment that the water taken from the most tranquil part of the lake, even after being agitated, contained in solution more than its own volume of carbonic acid gas, with a very small quantity of sulphuretted hydrogen." He added, that its temperature kept pretty constantly at 80 deg. Fahr., and that it is peculiarly fitted to afford nourishment to vegetable life. The banks of this Travertin lake are everywhere covered with reeds, lichens, confervæ, and various kinds of aquatic plants, which, as they grow and luxuriate, become gradually incrusted with crystals of calcareous matter. The lake, in fact, is called *Isoli Flottanti*, from the quantities of detached masses of vegetable matters floating about on its surface. The Anio, which receives the waters of this lake, incrusts the reeds, which grow on its banks, and the foam of the cascades at Tivoli form beautiful pendant stalactites. The rapid growth of the delta of the Tiber is attributed to the amount of the carbonate of lime that this river receives through the Anio from the steaming Lago di Zolfo. The river Anio holds an important place in the sanitary history of Rome, for this remarkable stream, before it gets polluted by the waters of the Lacus Albula, is pure, limpid, and wholesome to drink, and supplied the inhabitants of the Seven Hills with a delicious water, which was conducted to them by two aqueducts, one built by M. Curius Dentatus and Fulvius Flaccus, B.C. 271, and called the *Anio Vetus*. This received the water of the Anio about a mile above Tibur, and was forty-three miles in length. The other was between fifty-eight and sixty-two miles long, was called the *Anio Novus*, and had its origin on the banks of the natural river at a distance of forty-two miles from Rome. Such were the enormous works of a people, who were well aware of the necessity of a good supply of water to a great city; situated as the city was, in a volcanic district, the springs of which were tainted with sulphureous and other gases, they had but few resources if we except the yellow Tiber, and the fountains of Egeria and Mercury, which last were unable to supply the naturally increasing wants of a vast and thriving city. The aqueducts alone prove that the health of the citizen was considered of the highest importance to the state, whose rulers dispensed with no niggard hand, the greatest prophylactic against disease, an abundance of pure water, which, whilst it afforded the most wholesome beverage, cleansed the people and flushed the drains of their city.

We find from Lyell that the hills of Rome are capped by travertine and calcareous tufa, which enclose terrestrial shells, resembling those that now are seen in the gardens; fluviatile and lacustrine shells, identical with those found at the present day in the lakes and streams of the Campagna. On mounts Aventine, Vatican, and Capitoline, reeds and other vegetables are found incrusted with calcareous tufa, and intermingled with volcanic sand and pumice. The tusk of a mammoth has been discovered in this formation, filled in the interior with solid travertin. This formation, called the newer pliocene, rests partly on marine tertiary strata, which belong to the older pliocene era, and partly on volcanic tuff of later date. Their formation is supposed to have taken place in small lakes and marshes, which existed before the excavation of the valleys, which divide the Seven Hills of Rome, and they must originally have occupied the lowest hollows of the country, whereas we now find them placed upon the summit of hills about two hundred feet above the alluvial plain of the Tiber, which has flowed in its present chanuel ever since the building of Rome, since which scarcely any alterations in the geographical features of the country have taken place. Professor Lyell adds that when the marine tertiary strata of this district were formed, those of Montemario for instance, the Mediterranean was already inhabited by a large proportion of the existing species of crustacea. At a subsequent period, volcanic eruptions occurred, and tuffs were superimposed. The marine formation then emerged from the deep and supported lakes, wherein the fresh-water groups above described slowly accumulated, at a time when the mammoth abounded in the country. The valley of the Tiber was afterwards excavated by the hand of nature, and the adjoining hills assumed their present shape, and then a long interval may perhaps have elapsed before the first human settlers arrived. Thus we have evidence, concludes this learned writer, of a chain of events, all regarded as extremely recent by the geologist, but which, nevertheless, may have preceded for an immense series of ages a very remote era in the history of nations.*

The physical and geological character of the site of Rome has ever had such a remarkable effect upon its climate, and consequently upon the health of its inhabitants, that we are naturally led to present a sketch of this subject before entering upon any other.

*The Antient Climate of Rome.* Situated as Rome was, in the midst of an unhealthy district, its physicians had to contend against a host of fevers resulting from malaria. It is hard, therefore, to understand what Cicero† meant when he said, that Romulus had selected a "healthy site in the midst of a pestilential region." During the early part of the Roman history it appears, however, that the pestiferous exhalations were diminished by agriculture, and that so soon as this science declined, the insalubrity of the fertile plains increased. Again, when the city became immensely crowded with inhabitants, it was observed that fevers having their origin at the foot of the Seven Hills, seemed to have lost their power in affecting the population. The hills of Rome were not of sufficient height to give those who inhabited them a perfect immunity from the disease, who, at its acme, found that their neighbours escaped,—viz., the inhabitants of the district on which Tibur, Aricia, and Lannovium were built. The poor, of course, during the most fatal season, which was in the dog-days, were obliged to remain in the city, but the wealthier classes visited their villas in the neighbourhood until the virulence of an endemic had subsided.

With regard to the character of the meteorological phenomena generally observed throughout the seasons at Rome, we have but few facts before us. In the writings of the true poets, who generally took their images from nature, we often find valuable records which historians pass over as unworthy of their pens. Diseases of

---

* LYELL. Prin. of Geology, vol. iii, p. 138 and seq.

epidemic character were not, it appears, confined to the human subject, for we find Horace, in his earnest appeal to the Romans, in which he advises them to emigrate and go to the Fortunate Isles, distinctly drawing their attention to the fact that during the reign of the dog-star flocks are injured by disease, and that at one time they are deluged by the watery Eurus (easterly wind) and at another, the fertile fields are parched up for want of rain.*

With the winter, the *north* winds were frequently associated, and they sometimes blew very high. These winds are called by the moderns, *Tramontane,* they seldom continue more than three days together.

The *south* wind was always considered deleterious, and is noticed as occurring during autumn,

> "The southern blast in vain we fear,
> And autumn's life annoying airs
> With idle fears alarm."†

Perhaps this passage alludes to the *Sirocco,* (a south wind from the north of Africa,) which has a most deleterious effect upon invalids, and an enervating one even on the robust, especially the sensitive and plethoric.‡

*The west wind* was always a favourite, and accompanying spring, as the poets say, was as frequently extolled as the south wind was abused. Thus Horace§ alludes to the winds of spring, in his elegant epistle to Mæcenas, who wants the poet to visit him, but is told that his friend has retired to his Sabine farm to breathe the mountain air during the sickly season at Rome, and that after staying at the sea coast during the winter he would visit his patron in the spring.

> "When western (*zephyris*) winds, and the fast swallow bring
> The welcome tidings of returning spring."

The west winds were often instrumental, from ancient times, in blowing back the waters of the Tiber, and causing them to overflow their banks, and thus leave a quantity of saturated vegetable matter for the sun to act upon during the summer and autumn.

*The east wind,* (Eurus) has been mentioned above as being "watery"; perhaps Horace exaggerated its effects.

In speaking of this subject, in relation to modern Rome, a late writer, Sir James Clark, makes a highly interesting remark, and explains, to a great extent, the unhealthiness of the climate of Rome. He says, "one peculiarity of Rome is the *stillness* of its atmosphere, high winds being comparatively of rare occurrence." This calmness favours, without doubt, the accumulation, in the valleys and the plains around Rome, of those malarious gases which, in former times, as now, have ever proved so injurious to those enclosed within the walls of the city.

---

* Horat. Epodon, i, 6.

§ Horat. Ep. i, 7, 13.

Rome was sometimes visited by snow during the winter, and, probably, at times the Tiber itself was either partially or completely frozen over. Horace describes very beautifully the snow-belaboured woods of mount Soracte, the frozen waters of the rivers,* and the snow-clad plains of Alb.

According to Sir J. Clark, the mean temperature of the *winter* at Rome is 10° higher than London and 5° than Penzance. The mean annual temperature is also 10° higher than London and 8 higher than at Penzance. In *spring*, the mean temperature is 9° above that of London and 8° that of Penzance. Altogether, he says, that the climate of Rome is mild and soft, but rather relaxing and oppressive, still, he adds, it cannot be considered a damp climate.

*The Climate of Rome in Relation to Disease.* There is every reason to believe that Celsus, whilst writing, had before him the works of Hippocrates, for whole passages in his book can be traced to this source, literally translated into Latin: it is probable, however, that Celsus only admitted those general facts which were equally applicable to Greece and to Rome, using his own individual experience in all matters appertaining to the peculiarities in the effects of the climate of Rome, and the diseases of his fellow-citizens.

"The healthiest season is spring, and next to it ranks the winter: the summer, however, is more unhealthy, and the autumn by far the most dangerous time. Of the seasons generally, whether cold or hot, those are the healthiest that are of uniform temperature; the worst are those in which there are great vicissitudes of weather; whence it happens that the autumn affects so many. For there is heat during mid-day, whilst the nights, mornings, and evenings are cold." With regard to the calmness alluded to above, he thinks "that where there is uniformity of temperature calm (sereni) days are the healthiest, and that rainy days are better than those that are either foggy or cloudy: in winter it is best when the wind does not blow; in the summer, when there are gentle western (favonian) gales." Of the winds, he considered the westerly more healthy than either those from the east or the south; and he contrasts the salubrious qualities of winds coming from inland districts with those that sweep over the sea, (as the south wind, or sirocco, does).

As we have seen above, summer and autumn were considered the most unhealthy seasons in Rome, and modern observations will bear out the antient ones.

*Diseases of Antient Rome. Fever.* Celsus said that during summer fevers of several kinds, from the *continued* to the *tertian*, were prevalent; and that in autumn there arose others of an uncertain character, affecting the *spleen*. The *intermittent* character of this unwholesome visitor is sufficiently proved by the writings both of the antient and of the modern physicians. In our own country, in

* Hor. Car.

the fenny districts of Lincolnshire, Essex, and similarly situated localities in many other counties, we may almost daily see this disease or its effects. The Walcheren fever was the same; modified, indeed, as this fever is in every part of the world by climate, it still preserves its character, like the human race, however black, copper-coloured, or white, some members of it may be. What is the cause of these fevers? The reader has only to refer to the topographical position of Rome with regard to the plains and marshes of Latium, to find a solution to this query. The older physicians were well aware of the effects of marsh malaria. In his treatise on diseases of the spleen, which are intimately connected with intermittent fevers, Aretæus enumerates among the causes, "marshy places; brackish, bitter, and strong waters; and the autumnal season."* Galen,† who practised for some time at Rome, mentions that the exhalations from swamps are a frequent cause of fever, of what may be termed the *pernicious* kind, especially in the summer. This observation he has recorded in more than one place, in reference to the allusion made under the climate of ancient Rome, that in proportion as the population increased, so did malarious fevers disappear from the city. This has been partly accounted for by the fact that during the prosperity of the empire the plains were more assiduously cultivated, but that when they were let out for pasturage, and the grandeur of the Romans began to decline, fevers became more rife than ever. There is another reason assigned for the diminution of endemic diseases, viz., the simple fact that the increased heat of large cities produces a corresponding dryness, which two conditions being combined, have the effect of combating the attacks of the besieging malaria. Sir James Clark believes that a man may sleep all night in the Pontine marshes, provided that he keeps his windows closed and his chamber well heated. Persons in ill health, or who from luxury or any other cause have become enervated, are more prone than others to take the fever. Dyspeptics are particularly liable; can we then wonder that in the later years of Rome, when luxury, high feeding, constant bathing, late hours, and debaucheries of all kinds, were undermining that bodily and mental vigour, which had in the earlier and chaster periods of her history upraised this grand empire,—can we wonder that the gaseous enemy from without should so affect the humbled inhabitants of fallen Rome and devastate Campania, when that great crisis in the history of the world was brought about by the vigorous hand of the barbarian. Beyond running away from the disease, little else was done either by the state or the people themselves that could be called a sanitary regulation on this point.

During the *winter* the Romans were subject to *diseases of the eyes, hæmorrhages, boils, melancholia, insanity, neuralgic affections,*

---

* Aretæus, p. 112.

† Galen, vol. vii, p. 290; vol. xv, p. 121.

and *headaches.* The climate has ever been obnoxious to persons having an hæmorrhagic tendency, as well as to the subjects of hypochondriacism, and low, nervous depression. We find, also, that *paralysis* and *apoplexy* held a prominent place among the diseases treated of by Roman physicians. As in olden times, so is it now, for Rome is still unfit for those having a disposition to these affections. Celsus observed that paralysis and epilepsy occurred frequently during showery weather. Sir James Clark mentions that headaches are still common at Rome, especially among strangers. If from the first part of winter the southerly wind continued until the latter end of spring, those affected with fever were also attacked by alarming affections of the brain.*

With regard to the more chronic affections, such as subacute attacks of organs that had been left diseased by previous attacks of fever, Celsus remarked especially of the *liver* and *spleen,* that hot weather sets up inflammation in them.

*Consumptive cases* were mentioned by ancient authors, but were perhaps not of the true strumous character; there is no evidence to the contrary; at all events modern Rome is favourable to the *cure* of such cases. It will be also remembered by some, perhaps, that M. Louis pointed out a sort of antagonism between consumption and intermittent fever; if this really exists there may be found in the fact an answer to the question why pulmonary consumption, independent of other causes, is comparatively rare at Rome.

The *pulmonary cases,* which Celsus, Galen, and other authors record as having been induced and aggravated by the north winds, &c., were, in all probability, active inflammatory attacks of the lungs, which often proved fatal in consequence of the previously diseased state of the abdominal viscera consequent on ague. The above are a few of the more prominent diseases peculiar to the Roman climate. From these more general diseases it will be interesting to proceed to those whose virulence has gained for them the term of *pestilences,* and which from time to time appeared among the Romans, to their utter consternation and incalculable loss.

*Alfred Haviland.*

---

